# A new candidate oncogenic lncRNA derived from pseudogene WFDC21P promotes tumor progression in gastric cancer

**DOI:** 10.1038/s41419-021-04200-x

**Published:** 2021-10-02

**Authors:** Huaiping Cui, Zhaoyu Jiang, Shujie Zeng, Hao Wu, Zihao Zhang, Xiaobo Guo, Kangdi Dong, Jinshen Wang, Liang Shang, Leping Li

**Affiliations:** 1 Department of Gastrointestinal Surgery, Shandong Provincial Hospital, Cheeloo College of Medicine, Shandong University, Jinan, Shandong China; 2 Department of Gastrointestinal Surgery, Shandong Provincial Hospital Affiliated to Shandong First Medical University, Jinan, Shandong China; 3 Shandong Provincial Laboratory of Translational Medicine Engineering for Digestive Tumors, Shandong Provincial Hospital, Jinan, Shandong China

**Keywords:** Gastric cancer, Gastric cancer

## Abstract

As oncogenes and tumor suppressor genes, long non-coding RNAs (lncRNAs) regulate the biological behavior of gastric cancer (GC) cells such as proliferation, invasion, and metastasis through various signal pathways. At present, although numerous lncRNAs that significantly influence the development and progression of GC have been identified, a considerable number of them have not been found and studied yet. In this study, we identified a new lncRNA derived from pseudogenes WFDC21P, which have not been reported in any previous GC study. LncRNA WFDC21P was significantly upregulated in GC cells and tissues, and clinically associated with the pathological stages of advanced GC. WFDC21P promoted proliferation and metastasis of GC cells both in vitro and in vivo. LncRNA WFDC21P was directly bound to GTPase Ran and it promoted the activity of the Akt/GSK3β/β-catenin pathway. Forkhead Box P3 (FOXP3), as a transcription factor of WFDC21P, was directly bound to the promoter region and it positively regulated the transcription of WFDC21P. This finding may provide a novel biomarker and therapeutic target for GC.

## Introduction

Gastric cancer (GC) is the fifth most commonly diagnosed cancer (5.6%) and the fourth most common cause of cancer death (7.7%) in global cancer statistics 2020 [1]. As one of the most common malignant tumors [2, 3] in China, GC is highly malignant and invasive, and patients often die of its final outcomes [4]. Therefore, the studies on the molecular mechanisms of GC are particularly important, especially on those that are closely related to the malignant transformation, invasion, and metastasis of the tumor. The discovery of new molecules and targets makes a huge difference in the diagnosis and treatment of GC [5–7].

Long non-coding RNAs (lncRNAs) are a group of RNA molecules with more than 200 nucleotides in length; they lack the open reading frame (ORF) in molecular structure, which stops them from encoding proteins [8]. LncRNAs represent a quite high proportion in the whole genome and they are differently expressed in GC [9]. As oncogenes and tumor suppressor genes, lncRNAs regulate the biological behavior of GC cells such as proliferation, invasion, and metastasis through various signal pathways [10–12]. Although numerous lncRNAs that significantly influence the occurrence and progression of GC have been identified, a considerable number of lncRNAs have not been found and studied yet.

Previously, to identify the lncRNAs expression differences between GC tissues and normal gastric tissues (GEO database, GSE54835), we adopted a microarray platform containing probes for lncRNAs expressed from the human genomes in addition to a selected set of cancer metastasis-related lncRNAs. And we identified some lncRNAs, including C21orf96 and OR3A4, as new oncogenic molecules [13, 14]. Now, we identified a new lncRNA derived from pseudogenes WFDC21P (NR_030732.1, 621 nt in length) based on the expression differences between GC tissues and normal gastric tissues, which have not been reported in any previous study on GC.

In this study, we found that lncRNA WFDC21P was significantly upregulated in GC cells and tissues and associated with the pathological stages of advanced GC. Moreover, we discovered that WFDC21P promoted proliferation, invasion, and migration of GC cells both in vitro and in vivo. Mechanistically, we identified that the lncRNA WFDC21P was directly bound to GTPase Ras-related nuclear protein (GTPase Ran) and promoted the activity of Akt/GSK3β/β-catenin through RNA pulldown and RNA immunoprecipitation (RIP) assays. Forkhead Box P3 (FOXP3) was identified as a positive transcriptional factor of WFDC21P and FOXP3 knockdown inhibited the expression of WFDC21P. Collectively, we found that WFDC21P was an oncogene and investigated its biological function and regulatory mechanism in GC.

## Results

### LncRNA WFDC21P is highly expressed in human GC cells and tissues

The microarray analysis was performed in the previous study, and some highly expressed lncRNAs in GC tissues were found and verified, such as OR3A4, LOC84740, and FCGR1C et al. [14]. In the present study, we continue to explore novel oncogenic lncRNAs based on the microarray data.

**Fig. 1:**
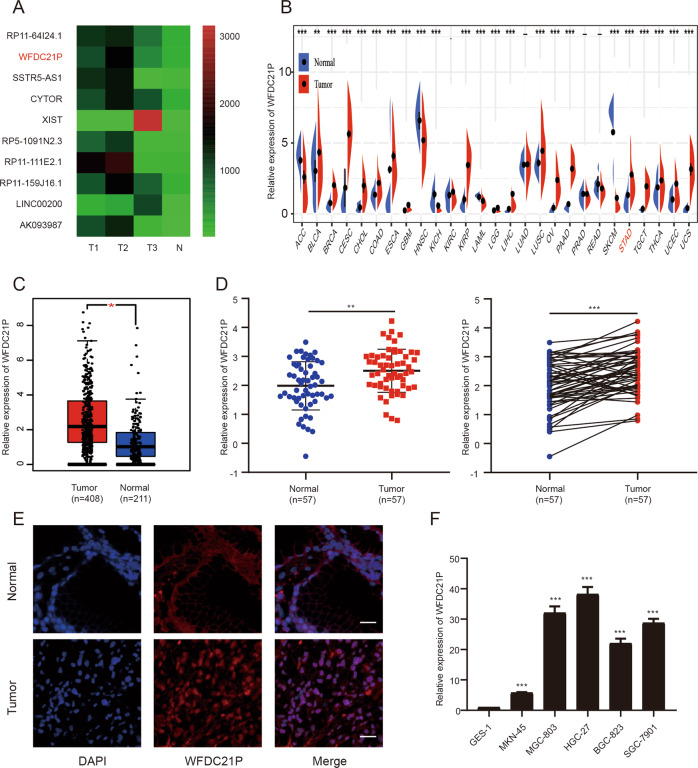
LncRNA WFDC21P is upregulated in GC. **A** Heat map of upregulated lncRNAs. **B** Relative expression of WFDC21P in multiple human Tumors from TCGA. **C** Relative expression of WFDC21P in GC tissues and normal tissues from TCGA. **D** Relative expression of WFDC21P in GC tissues and paired normal tissues was detected by qRT-PCR (*n* = 57). **E** Expression and localization of WFDC21P in GC tissue and normal tissue were detected by FISH assays. Scale bar, 20 μm. **F** Relative expression of WFDC21P in GC cell lines and GES-1 was detected by qRT-PCR. Data were shown as mean ± SD. (Student’s *t*-test and paired-samples *t*-test, *p < 0.05, **p < 0.01, ***p < 0.001).

To narrow the scope and facilitate the subsequent experiments of gene knockdown or overexpression, we artificially set the fold change >6 and the length <3000 nucleotides as screening criteria. Based on the above restriction, 44 lncRNAs were screened out (the details were listed in Supplementary Table 1) and 10 of them were selected for further bioinformatics analysis and qRT-PCR validation in a small number of tissue samples (Fig. 1A). The results showed that lncRNA WFDC21P, CYTOR, XIST, and AK093987 were significantly upregulated in GC according to the Cancer Genome Atlas (TCGA) database (Supplementary Fig. A), and lncRNA WFDC21P was most significantly upregulated in 12 GC tissues compared with matched normal tissues (*p* = 0.005, Supplementary Fig. B). Moreover, the TCGA database showed that lncRNA WFDC21P was significantly upregulated in 18 out of 27 tumors, including bladder cancer, breast cancer, colon cancer, esophageal cancer, pancreatic cancer, and GC et al. (Fig. 1B, C), offering the possibility for WFDC21P to be a pan-oncogene. In addition, we detected the expression of WFDC21P in 57 cases of GC tissues and matched normal tissues via qRT-PCR. The results showed that the expression of WFDC21P in GC tissues was significantly higher than that in normal tissues (Fig. 1D), which was consistent with the results of microarray data and the TCGA database. Clinicopathological analysis indicated that WFDC21P expression was positively correlated with GC tumor invasion, lymph node metastasis, TNM stage, and tumor size (Table 1). Fluorescence in situ hybridization (FISH) assay also proved that the expression of WFDC21P in GC tissues, especially in the tissue nucleus, was significantly higher than that in normal tissues (Fig. 1E).

**Table 1:** Correlation between lncRNA WFDC21P expression and clinicopathological characteristics in 57 GC patients.

Parameters	Cases	WFDC21P expression		*p*-value
		Low	High	
Total	57	28	29	
Age				0.839
<60	17	8	9	
≥60	40	20	20	
Gender				0.325
Male	35	19	16	
Female	22	9	13	
Tumor invasion				0.044^a^
T1–T2	12	9	3	
T3–T4	45	19	26	
Lymph node metastasis				0.007^a^
N0	17	13	4	
N1–N3	40	15	25	
TNM stage				0.022^a^
I–II	22	15	7	
III	35	13	22	
Tumor size				0.012^a^
<5 cm	25	17	8	
≥5 cm	32	11	21	

^a^Statistically significant.

Furthermore, WFDC21P expression was examined in GC cell lines and the normal gastric epithelium cell line GES-1 with qRT-PCR, and the results showed that the expression of WFDC21P in MKN-45, MGC-803, HGC-27, BGC-823, and SGC-7901 cell lines was significantly higher than that in GES-1 cell line (Fig. 1F). Among GC cell lines, MKN-45 showed the lowest expression and HGC-27 showed the highest expression of WFDC21P, so the two cell lines were selected for further study.

### LncRNA WFDC21P promotes proliferation, migration, and invasion of GC cells in vitro

**Fig. 2:**
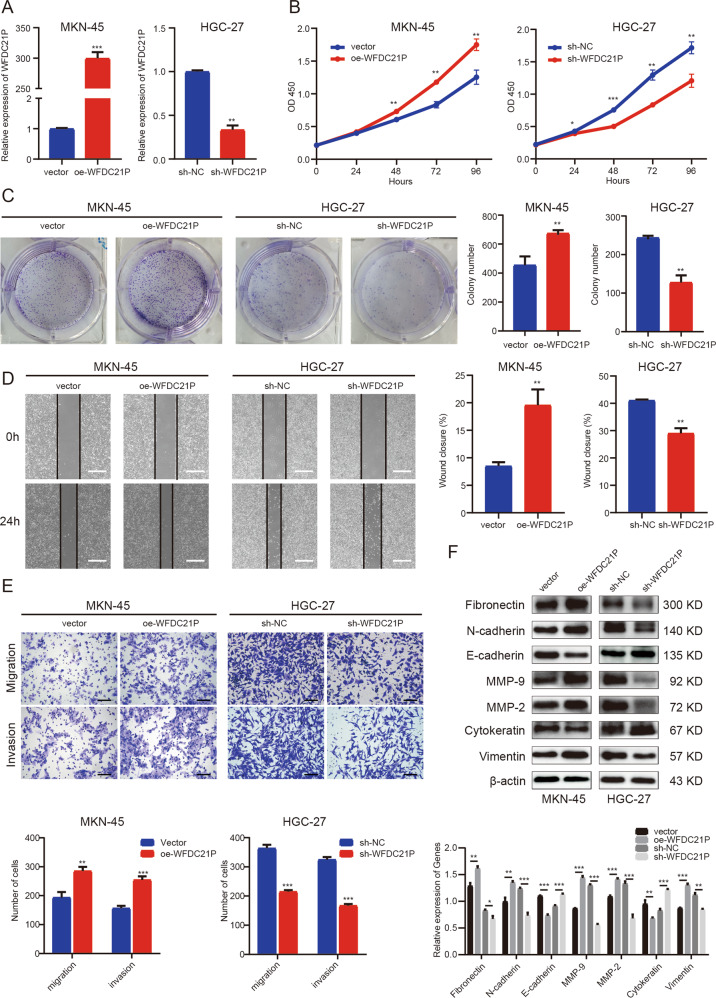
LncRNA WFDC21P promotes proliferation, migration, and invasion of GC cells in vitro. **A** Efficiencies of overexpression and knockdown of WFDC21P were detected by qRT-PCR. **B**, **C** CCK-8 and colony formation assays were performed to detect the proliferation of oe-WFDC21P or vector-transfected MKN-45 cells and sh-WFDC21P or sh-NC transfected HGC-27 cells. **D**, **E** Scratch and transwell assays were performed to detect the migration and invasion of oe-WFDC21P or vector-transfected MKN-45 cells and sh-WFDC21P or sh-NC transfected HGC-27 cells. The white scale bar, 500 μm; the black scale bar, 100 μm. **F** Western blot was performed to detect the expressions of EMT-related proteins in oe-WFDC21P or vector-transfected MKN-45 cells and sh-WFDC21P or sh-NC transfected HGC-27 cells. Data were shown as mean ± SD. (Student’s *t*-test, *p < 0.05, **p < 0.01, ***p < 0.001). NC means negative control, and oe means overexpression.

To further investigate the biological role of WFDC21P in GC cells, lentivirus vectors with stable overexpression and knockdown of WFDC21P were conducted and successfully transfected into MKN-45 and HGC-27 cells (the two cell lines with the lowest expression and the highest expression of WFDC21P, respectively). The transfection efficiency was verified by qRT-PCR (Fig. 2A). Cell counting kit-8 (CCK-8) proliferation assay results indicated that the overexpression of WFDC21P significantly accelerated the growth of MKN-45 cells, whereas the knockdown of WFDC21P significantly inhibited the growth of HGC-27 cells (Fig. 2B). Colony formation assay results indicated that the overexpression of WFDC21P significantly promoted colony formation in MKN-45 cells, while the knockdown of WFDC21P generated opposite effects in HGC-27 cells (Fig. 2C). The two experiments demonstrated that WFDC21P promoted the proliferation of GC cells.

Afterward, scratch assays and transwell assays were carried out to examine the effects of WFDC21P on migration and invasion of GC cells. Scratch assay results indicated that the scratch healing was significantly promoted by the overexpression of WFDC21P in MKN-45 cells, while it was significantly inhibited by the knockdown of WFDC21P in HGC-27 cells (Fig. 2D). Transwell assay results also indicated that the overexpression of WFDC21P significantly promoted the migration and invasion of MKN-45 cells, whereas the knockdown of WFDC21P significantly inhibited the migration and invasion of HGC-27 cells (Fig. 2E). Moreover, the overexpression of WFDC21P significantly promoted the expression of epithelial-mesenchymal transition (EMT) related proteins in MKN-45 cells, whereas the knockdown of WFDC21P significantly inhibited the expression of EMT-related proteins in HGC-27 cells (Fig. 2F). These experiments suggested that WFDC21P promoted the migration and invasion of GC cells.

### Subcellular localization of lncRNA WFDC21P

**Fig. 3:**
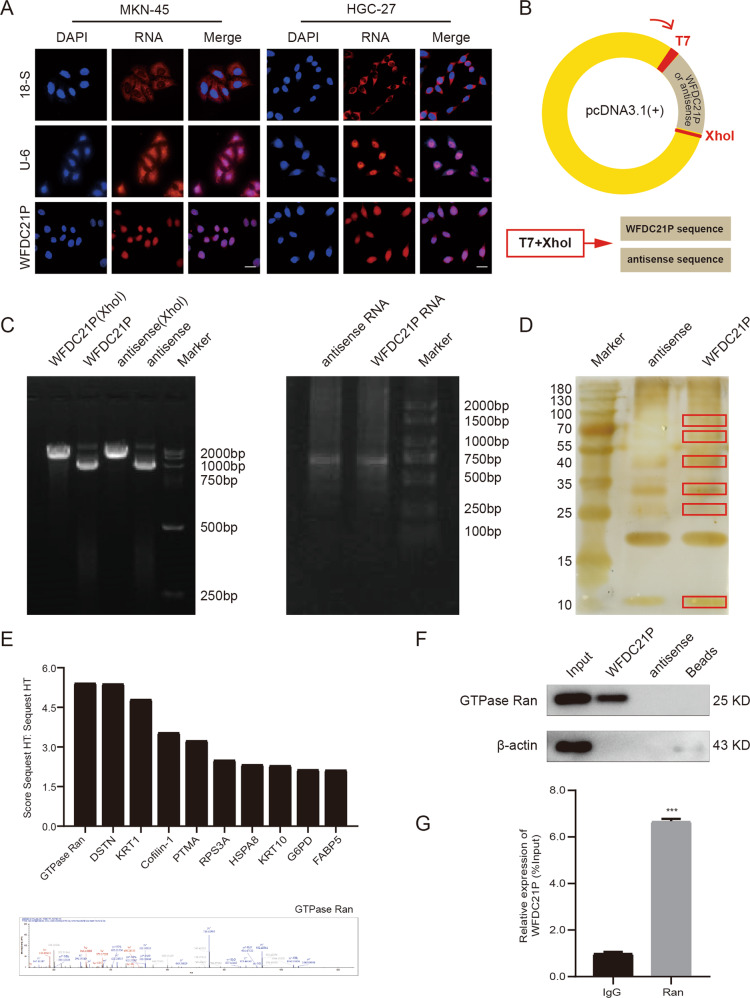
LncRNA WFDC21P binds to GTPase Ran protein. **A** Subcellular localization of WFDC21P in MKN-45 and HGC-27 cells was detected by FISH assays. Scale bar, 20 μm. **B** Pattern diagram of the plasmid, which contained WFDC21P or its antisense sequence. **C** WFDC21P or its antisense sequence was linearized with the enzyme digestion of XhoI and T7 sites, transcribed, and purified in vitro. **D** Silver staining assay was performed to detect the proteins obtained by the pulldown assay in HGC-27 cells. **E** The top 10 proteins in perceived credibility were identified by mass spectrometry and GTPase Ran was identified as a WFDC21P-binding protein. **F** Western blot and another independent RNA-pulldown assay were conducted to confirm the interaction of WFDC21P and Ran in HGC-27 cells. **G** RIP assay was performed to detect the Ran–WFDC21P interaction in HGC-27 cells. Data were shown as mean ± SD. (Student’s *t*-test, ***p < 0.001).

The subcellular localization of lncRNAs is closely related to their function. To determine the subcellular localization of WFDC21P, we performed the FISH assay using U6 and 18S as cytoplasmic and nuclear controls, respectively. The result showed that WFDC21P was widely distributed but mainly located in the nucleus of HGC-27 and MKN-45 cells (Fig. 3A), which was consistent with the result of the FISH assay in GC tissues (Fig. 1E).

### LncRNA WFDC21P directly binds to GTPase Ran protein

It was reported that lncRNAs could promote tumor progression through directly binding to tumor-related proteins [15–17]. In the present study, an RNA-pulldown assay was used to find the proteins binding to WFDC21P and the HGC-27 cell line with the highest expression of WFDC21P was selected as the target cell line. Firstly, we synthesized the exogenous plasmid which contained WFDC21P or its antisense sequence (Fig. 3B), and then we linearized the RNA sequences and transcribed them in vitro (Fig. 3C). Finally, the biotin-labeled RNA was incubated with the protein lysate of HGC-27 cells, and the proteins were identified by 12.5% SDS-PAGE and silver staining (Fig. 3D). The two protein solutions were analyzed by mass spectrometry and the result indicated that GTPase Ran was the highest-scoring WFDC21P RNA-binding protein (Fig. 3E and Supplementary Table 2). To further verify our results, western blotting and another independent RNA-pulldown assay were conducted to confirm the interaction of WFDC21P and Ran (Fig. 3F). Besides, the Ran–WFDC21P interaction was also confirmed by RIP assay (Fig. 3G).

### Ran is regulated by WFDC21P and promotes invasion and metastasis in GC cells

**Fig. 4:**
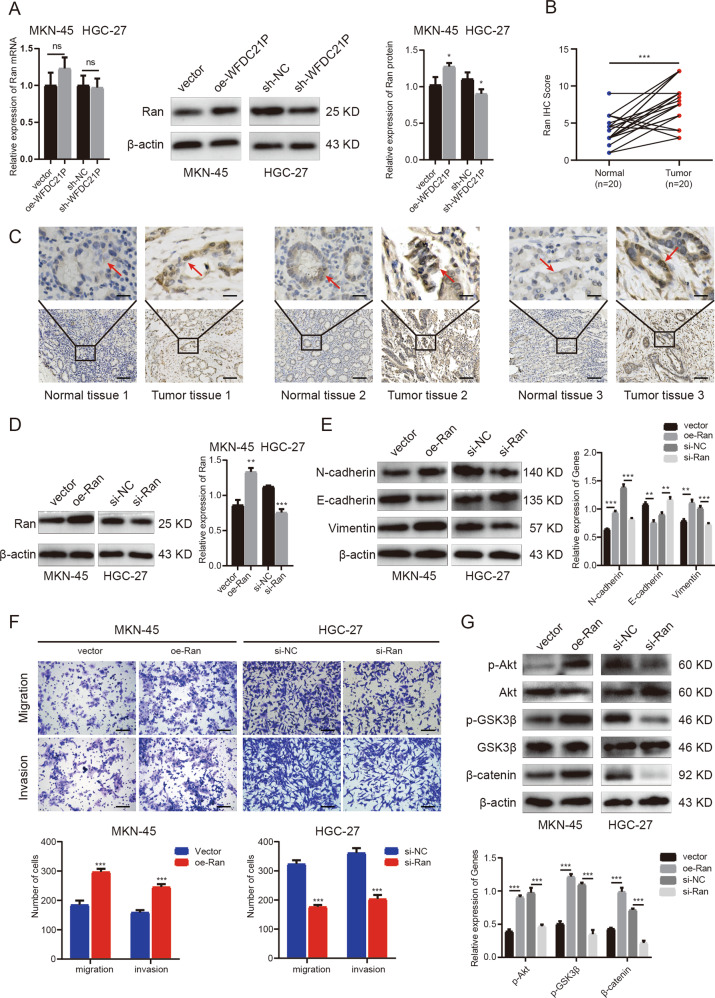
Ran promotes invasion and metastasis and activates Akt/GSK3β/β-catenin pathway in GC cells. **A** WFDC21P regulated the expression of Ran protein rather than mRNA in GC cells. **B** IHC scores of Ran in GC tissues and paired normal tissues (*n* = 20). **C** IHC staining of Ran in GC tissues and paired normal tissues. Scale bar, 20 μm (above), 100 μm (below). **D** Efficiencies of overexpression and knockdown of Ran were detected by western blot. **E** Western blot was performed to detect the expressions of EMT-related proteins in oe-Ran or vector-transfected MKN-45 cells and si-Ran or si-NC transfected HGC-27 cells. **F** Transwell assays were performed to detect the migration and invasion of oe-Ran or vector-transfected MKN-45 cells and si-Ran or si-NC transfected HGC-27 cells. Scale bar, 100 μm. **G** Western blot was performed to detect the expressions of Akt/GSK3β/β-catenin pathway proteins in oe-Ran or vector-transfected MKN-45 cells and si-Ran or si-NC transfected HGC-27 cells. Data were showed as mean ± SD. (Student’s *t*-test and paired-samples *t-*test, ns means no significant difference, *p < 0.05, **p < 0.01, ***p < 0.001).

Through the above RNA-pulldown assay and RIP assay, we confirmed that lncRNA WFDC21P was directly bound to Ran, but the regulatory relationship between them needed to be further explored. Therefore, we analyzed the Ran expression in the WFDC21P overexpressed MKN-45 cells and the WFDC21P downregulated HGC-27 cells by qRT-PCR and western blot. The qRT-PCR results showed that the overexpression and knockdown of WFDC21P had no significant effect on the mRNA expression of Ran in HGC-27 (*p* = 0.791) and MKN-45 (*p* = 0.158) cells (Fig. 4A left). The results of the western blot showed that the overexpression of WFDC21P promoted the expression of Ran (*p* = 0.021), and the knockdown of WFDC21P inhibited the expression of Ran (*p* = 0.033) (Fig. 4A right). It suggested that WFDC21P may regulate the expression of Ran at the protein level rather than the mRNA level.

Ran is a small GTP binding protein belonging to the Ras superfamily, and considered to be a key player in tumor metastasis [18, 19]. Ran was reported to activate the EMT process and various metastasis-related signaling pathways, such as the Akt pathway, Ras pathway, and β-catenin pathway [20, 21]. The oncogenic functions of Ran have been confirmed in colorectal cancer, breast cancer, ovarian cancer, and other solid tumors [22–25]. In GC, Ran was found upregulated and expected to be an immune recognition site molecule [26].

In the present study, we examined the expression of Ran in 20 cases of GC tissues and matched normal tissues by immunohistochemistry (IHC) staining. The result of IHC staining showed that the expression of Ran in GC tissues was significantly higher than that in normal tissues and it was mostly distributed in the nucleus in GC tissues (Fig. 4B, C), which was consistent with the previous study performed by Azuma et al. [26]. To explore whether Ran also plays an important role in GC metastasis, we knocked down and overexpressed Ran in HGC-27 and MKN-45 cells (Fig. 4D). Western blot assays showed that the overexpression of Ran significantly increased the expression of N-cadherin and Vimentin and decreased the expression of E-cadherin in MKN-45 cells, while the knockdown of Ran showed just the opposite result in HGC-27 cells (Fig. 4E). These results indicated that Ran could activate the EMT process in GC cells. Transwell assays results also indicated that the overexpression of Ran significantly promoted the migration and invasion of MKN-45 cells, while the knockdown of Ran inhibited the migration and invasion of HGC-27 cells (Fig. 4F). To explore the downstream mechanism of Ran, we examined the molecular changes in multiple pathways. We found that overexpression of Ran could increase the expression of p-Akt and p-GSK3β, thus promoting the activation of Akt/GSK3β/β-catenin metastasis-related signaling pathway in MKN-45 cells, while knockdown of Ran inhibited the pathway in HGC-27 cells (Fig. 4G), which was consistent with the previous study [21]. These experiments demonstrated that Ran promoted invasion and metastasis by activating Akt/GSK3β/β-catenin pathway in GC cells.

### LncRNA WFDC21P promotes GC cells invasion and metastasis and activates Akt/GSK3β/β-catenin pathway by targeting Ran

**Fig. 5:**
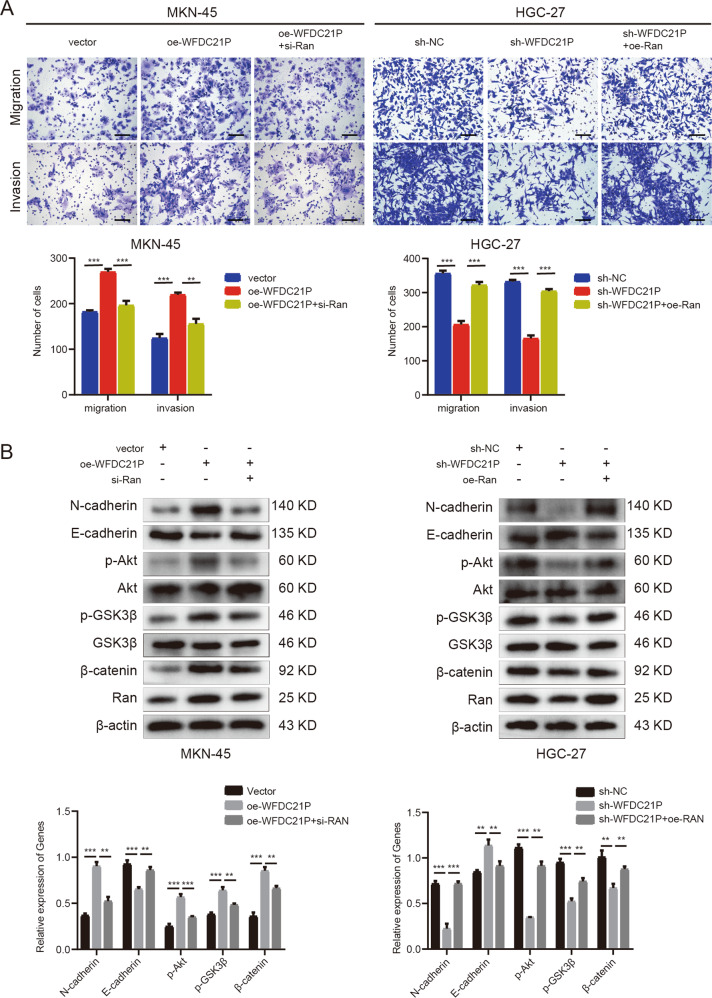
LncRNA WFDC21P promotes GC cells invasion and metastasis and activates Akt/GSK3β/β-catenin pathway by targeting Ran. **A** Transwell rescue assays were performed to detect the migration and invasion of oe-WFDC21P, vector or oe-WFDC21P + si-Ran transfected MKN-45 cells and sh-WFDC21P, sh-NC or sh-WFDC21P + oe-Ran transfected HGC-27 cells. Scale bar, 100 μm. **B** Western blot was performed to detect the expressions of N-cadherin, E-cadherin and Akt/GSK3β/β-catenin pathway proteins in oe-WFDC21P, vector or oe-WFDC21P + si-Ran transfected MKN-45 cells and sh-WFDC21P, sh-NC or sh-WFDC21P + oe-Ran transfected HGC-27 cells. Data were shown as mean ± SD. (Student’s *t*-test, **p < 0.01, ***p < 0.001).

To verify whether WFDC21P could promote the invasion and metastasis of GC cells by targeting Ran, the rescue experiments were designed. Transwell assays showed that si-Ran reversed the invasion and metastasis promoting effects induced by overexpression of WFDC21P in MKN-45 cells, whereas the oe-Ran counteracted the suppressing effects induced by knockdown of WFDC21P in HGC-27 cells (Fig. 5A). The experiments above have proved that Ran promoted the EMT process and Akt/GSK3β/β-catenin pathway (Fig. 4D, F). And we also found that overexpression of WFDC21P promoted the EMT process and activated the Akt/GSK3β/β-catenin pathway while knockdown of WFDC21P showed the contrary results. Simultaneously, the effects of the knockdown or the overexpression of WFDC21P on the EMT process and the Akt/GSK3β/β-catenin pathway were reversed by Ran overexpression or silence respectively (Fig. 5B). Collectively, these results demonstrated that WFDC21P promotes GC cells invasion and metastasis and activates Akt/GSK3β/β-catenin pathway by targeting Ran.

### WFDC21P is activated by transcription factor FOXP3

**Fig. 6:**
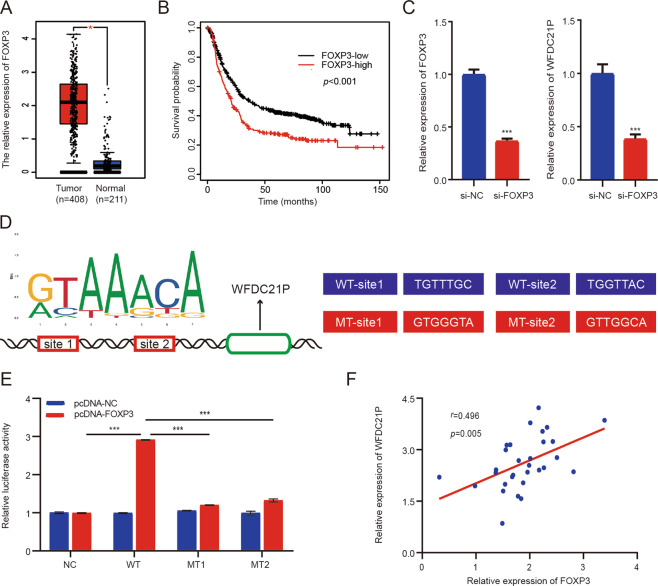
FOXP3 was a transcription factor of WFDC21P and positively regulated the expression of lncRNA WFDC21P. **A** Relative expression of FOXP3 in GC tissues and normal tissues from TCGA. **B** The survival curve of FOXP3 in GC patients based on K-M plotter database (http://www.kmplot.com). (*p* < 0.001) **C** The knockdown of FOXP3 led to the significant downregulation of WFDC21P expression detected by qRT-PCR. **D** Recognition bases of FOXP3, the two binding sites of FOXP3 to the promoter region of WFDC21P, and the sequences of the WT and MT for luciferase assay verification were shown. **E** The luciferase activity of MT1 and MT2 transfection groups both significantly decreased compared with the WT transfection group detected by luciferase reporter assay. **F** FOXP3 had a trend of co-expressed with WFDC21P in 30 GC tissue samples detected by qRT-PCR (*p* = 0.005). Data were shown as mean ± SD. (Student’s *t*-test and Pearson’s correlation analysis, ***p < 0.001). WT means wild-type, and MT means mutant-type.

To explore the upstream regulatory molecules of WFDC21P, we used the Promo (alggen.lsi.upc.es) and Jaspar databases (jaspar.genereg.net) to identify its potential transcription factors, and FOXP3 was selected as a possible target considering that its oncogenic functions had been confirmed in a previous GC study [27–29]. According to the TCGA database, FOXP3 is upregulated and associated with poor prognosis in GC (Fig. 6A, B). Subsequently, si-FOXP3 and si-NC were used to transfect HGC-27 cells, and the qRT-PCR assay result showed that the knockdown of FOXP3 led to the significant downregulation of WFDC21P expression (Fig. 6C). Further, we used the Jaspar database to explore the potential binding sites between FOXP3 and the promoter region of WFDC21P, and the top 10 binding sites with the highest score were shown in Supplementary Table 3. Next, we selected the two binding sites with the highest score (site1:710-716, site2:1179-1185) and mutated them for subsequent luciferase reporter assay verification (Fig. 6D). Luciferase reporter assay showed that compared with the wild-type (WT) group, the luciferase activity of mutant-type 1 (MT1) and MT2 groups both significantly decreased (Fig. 6E). Besides, 30 GC tissue samples were used to detect the expression of FOXP3 and WFDC21P, and the results showed that they had a trend of co-expressed (Fig. 6F). Collectively, these data demonstrated that FOXP3 directly bound to the promoter region of WFDC21P and positively regulated the expression of lncRNA WFDC21P.

### Knockdown of WFDC21P inhibits the growth and lung metastasis of GC in vivo

**Fig. 7:**
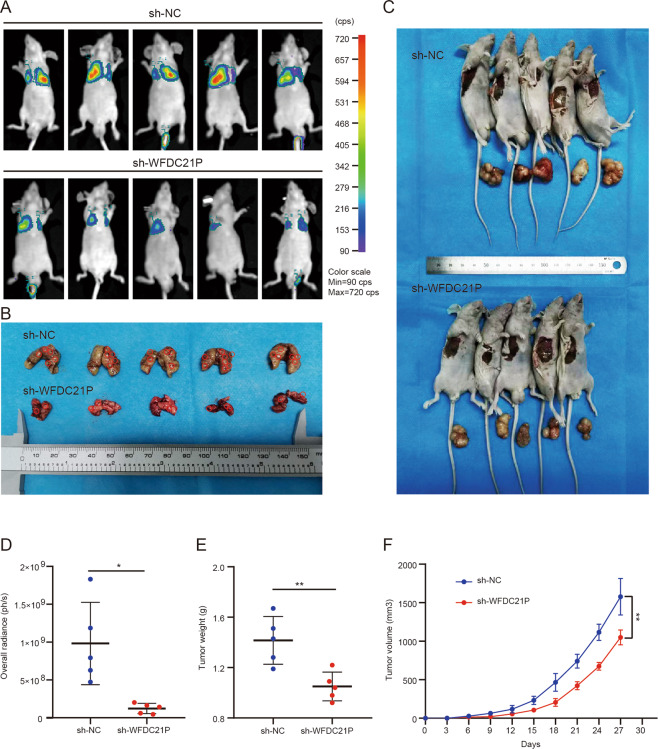
Knockdown of WFDC21P inhibits the growth and lung metastasis of GC cells in nude mice. **A** Bioluminescence images of tumors in nude mice were shown at 4 weeks after tail vein injection in sh-NC and sh-WFDC21P groups (*n* = 5 mice per group). **B** The lungs of nude mice were dissected at 4 weeks after tail vein injection in sh-NC and sh-WFDC21P groups. **C** The tumors of nude mice were dissected at 4 weeks after right flank injection in sh-NC and sh-WFDC21P groups (*n* = 5 mice per group). **D** The overall radiances of lung metastatic tumors in the sh-WFDC21P group significantly decreased compared with the sh-NC group. **E** The tumor weights in the sh-WFDC21P group significantly decreased compared with the sh-NC group. **F** The tumor volumes in the sh-WFDC21P group significantly decreased compared with the sh-NC group. Data were shown as mean ± SD. (Student’s *t-*test, *p < 0.05, **p < 0.01).

To further explore the effects of WFDC21P in vivo, lung metastasis and subcutaneous tumorigenesis models were constructed. In the metastasis model, luciferase-labeled HGC-27 cells (2 × 10^6^, 150 μl) that stably express sh-WFDC21P and their controls were injected into nude mice via tail vein. The results showed that the luminescence intensities in the lungs were significantly lower in mice in the sh-WFDC21P group than in mice in the control group, and the number of metastatic nodules and lung volumes in the sh-WFDC21P group were significantly lower than that in the control (Fig. 7A, B, D). In the tumorigenesis model, luciferase-labeled HGC-27 cells (5 × 10^6^, 150 μl) that stably express sh-WFDC21P and their controls were injected into the right flanks of nude mice. The size of the tumor was measured every 3 days. The results showed that the size and weight of the tumors were significantly decreased in the sh-WFDC21P group than those in the control group (Fig. 7C, E, F). These data demonstrated that knockdown of WFDC21P could inhibit the growth and lung metastasis of GC in vivo.

## Discussion

In the present study, we identified a long non-coding RNA from the pseudogene WFDC21P, which serves as an oncogene in GC. Firstly, we found lncRNA WFDC21P was significantly upregulated in GC and positively correlated with GC tumor invasion, lymph node metastasis, TNM stage, and tumor size. Besides, the results of cell experiments and nude mice experiments showed that lncRNA WFDC21P could promote the growth, invasion, and metastasis of GC cells in vitro and in vivo. In addition, it is found that GTPase Ran protein was directly bound to lncRNA WFDC21P by RNA-pulldown and RIP assays and GTPase Ran promoted GC cells metastasis via regulating EMT process and Akt/GSK3β/β-catenin pathway. Furthermore, we confirmed that lncRNA WFDC21P promoted GC cells metastasis and activates Akt/GSK3β/β-catenin pathway by targeting Ran with rescue experiments. Finally, FOXP3 was found as the transcription factor of WFDC21P, and it positively regulated the expression of lncRNA WFDC21P. Given the above-mentioned findings, the results suggested that lncRNA WFDC21P was a new candidate oncogene in human GC.

In tumors, the subcellular localization of lncRNAs is closely related to their functions. In this study, it was found that lncRNA WFDC21P was mainly located in the nucleus both in GC tissues and cell lines by FISH assays and that GTPase Ran was also mainly located in the nucleus of GC tissues by IHC staining. RNA-pulldown assay, RIP assay, and western blot assay were used to confirm that lncRNA WFDC21P was directly bound to GTPase Ran, and regulated the expression of Ran protein. Moreover, we found that Ran can promote the migration and invasion of GC cells. These results indicated that the oncogenic function of lncRNA WFDC21P in GC was partly mediated by RNA–protein interaction, which was one of the reported acting modes of lncRNAs. For example, it was reported that lncRNA MALAT1 could bind to and inactivate the pro-metastatic transcription factor TEAD to suppress breast cancer metastasis [15]. Besides, it was reported that lncRNA AGPG could bind to and stabilize PFKFB3 to promote cell proliferation in esophageal squamous cell carcinoma [30].

In addition to exploring the downstream regulatory mechanism of WFDC21P, we also investigated the causes of high expression of WFDC21P in GC. As reported, lncRNAs can be activated or inhibited by transcription factors. For example, the transcription of lncRNA LINRIS could be inhibited by GATA3 in CRC cells [31]. TEAD4 has also been reported to directly bind to the promoter region of MNX1-AS1 and stimulate the transcription of lncRNA MNX1-AS1 [32]. As a transcription factor, FOXP3 was reported to be upregulated in GC and promoted the proliferation and metastasis of GC cells [27–29]. In the present study, we found that FOXP3, as a transcription factor of WFDC21P, directly bound to the promoter region and positively regulated the transcription of WFDC21P, providing a reasonable explanation for the high expression and oncogenic role of WFDC21P in GC.

In conclusion, we identified a new candidate oncogenic lncRNA WFDC21P that promotes tumor progression through WFDC21P/Ran/Akt/GSK3β/β-catenin axis and that is positively regulated by transcription factor FOXP3 in GC, which may provide a novel biomarker and therapeutic target for GC. However, the specific mechanism of how WFDC21P affects Ran protein has not been clarified, which is a limitation of this study. Besides, further studies are needed to investigate how WFDC21P interacts with other proteins and how it is regulated by other transcription factors.

## Materials and methods

### Tissue specimens

Fifty-seven pairs of GC and matched adjacent normal gastric tissues were obtained from patients who underwent gastrectomy in the Shandong Provincial Hospital affiliated to Shandong University from 2015 to 2017. None of the patients had received neoadjuvant therapy before the operation. All tissue samples were stored in liquid nitrogen and briefly stored in a −80 °C refrigerator when being processed. All patients were informed consent and the study was approved by the Committee for Ethical Review of Research involving Human Subjects of Shandong Provincial Hospital.

### Cell lines

The GC cell lines MKN-45, MGC-803, HGC-27, BGC-823, SGC-7901, and the normal gastric epithelium cell line GES-1 were obtained from the Culture Collection of Chinese Academy of Sciences (Shanghai, China). The HEK-293 cell was obtained from the American Type Culture Collection (ATCC). All GC cell lines and GES-1 were cultured in RPMI-1640 medium (Gibco, NY, USA) and HEK-293 cell was cultured in DMEM medium (Gibco). Both media were supplemented with 10% fetal bovine serum (FBS, Gibco), and 1% penicillin–streptomycin (Gibco). The cells were maintained in a 5% CO_2_ incubator at 37 °C. All cells were verified by short tandem repeat profiling and tested negative for mycoplasma contamination.

### LncRNA microarray and data analysis

Microarray analysis was performed via the Agilent Array platform (Agilent Technologies, Santa Clara, CA, USA). The study was performed according to the previously established protocol [14].

### RNA extraction and qRT-PCR

The total RNA of tissues and cell lines was extracted with Trizol reagent (Invitrogen, CA, USA). Reverse transcription was performed in a 10 μl reaction system using Evo M-MLV RT Premix (Accurate Biotechnology, Hunan, China) according to the manufacturer’s protocol. The cDNA was amplified by real-time PCR using SYBR Green Pro Taq HS Premix (Accurate Biotechnology) and detected by the LightCycler 480 system (Roche Diagnostics, Basel, Switzerland). The 2^−ΔΔCt^ method was used to determine the relative quantification of RNA expression compared to internal control β-actin, and each sample was repeated three times. The primers were listed in Supplementary Table 4.

### Fluorescence in situ hybridization (FISH)

The FISH assay was conducted to detect the subcellular localization of lncRNA WFDC21P in GC tissues and normal tissues as well as in GC cell lines MKN-45 and HGC-27. Cy3 labeled lncRNA WFDC21P probe was designed and synthesized (GenePharma, Shanghai, China) and hybridize overnight with the cells or tissue sample to be tested based on the manufacturer’s instructions. The 18S probe (GenePharma) was used as the control for cytoplasmic localization, and the U6 probe (GenePharma) was used as the control for nuclear localization. The subcellular localization of lncRNA WFDC21P in the samples was observed by the confocal microscope.

### Cell transfection

The overexpression and knockdown recombinant lentivirus of lncRNA WFDC21P (oe-WFDC21P and sh-WFDC21P) and the negative control (sh-NC and vector) were designed and synthesized by Genomeditech (Shanghai, China). Small interfering RNAs against Ran (si-Ran) and FOXP3 (si-FOXP3) and the negative control (si-NC), and the plasmid of Ran (oe-Ran) were designed and synthesized by Genomeditech. Lipofectamine 3000 reagent (Invitrogen) was used as the transfection reagent according to the manufacturer’s protocol. Western blot and qRT-PCR were adopted to evaluate the transfection efficiency. All interfering sequences were listed in Supplementary Table 5.

### Cell counting kit-8 proliferation assay

Lentivirus-transfected cells were plated on 96-well plates with a density of 3000 cells per well, the proliferation of cells was detected by CCK-8 (Kumanoto, Japan) at 24, 48, 72, 96 h based on the manufacturer’s instructions. After being incubated at 37 °C for 2 h, the absorbance value of each well at 450 nm was measured. Each sample was repeated three times.

### Colony formation assay

Lentivirus-transfected cells were plated on 6-well plates with a density of 800 cells per well. The plates were maintained in a 5% CO_2_ incubator at 37 °C for 2 weeks. Then, the cells were washed twice with PBS after discarding the medium, fixed with paraformaldehyde for 30 min, and stained with crystal violet reagent for 30 min. Finally, the number of colonies was counted. Each sample was repeated three times.

### Scratch assay

Lentivirus-transfected cells were plated on 6-well plates to form a monolayer overnight, and then the monolayer was scratched with a 200 µl pipette tip. The floating cells were washed away by PBS, and the serum-free medium was added to the well and cultured in a 5% CO_2_ incubator at 37 °C for 24 h. The scratches were photographed at 0 and 24 h, and the areas and the widths were measured by the Image J software (NIH, Bethesda, Maryland, USA). Each sample was repeated three times.

### Transwell migration and invasion assays

The transwell chambers (Corning, NY, USA) with polycarbonate membranes of 8-μm pore were used in the migration and invasion assays. In the migration assay, the lentivirus-transfected MKN-45 cells or the HGC-27 cells (4 × 10^4^) were suspended in a 200 µl serum-free medium and planted in the upper chamber; medium containing 10% FBS was added to the lower chamber as the chemoattractant. After incubation for 24 h, cells on the upper chamber were completely scraped by cotton swabs, and the cells on the lower surface of the membrane were fixed with paraformaldehyde for 30 min, stained with crystal violet for 30 min, and photographed under a microscope (Olympus, Tokyo, Japan) at ×200 magnification. In the invasion assay, the upper chamber was coated with pre-diluted matrigel mix (3 mg/ml) (BD, NJ, USA) for 1 h at 37 °C before cells were added to the upper chamber. Then the lentivirus-transfected MKN-45 cells or the HGC-27 cells (1 × 10^5^) were suspended in a 200 µl serum-free medium and added to the upper chamber. The following steps were the same as migration assay. The invasion or migration of the GC cells was evaluated by the number of cells in 5 random fields using the Image J software (NIH, Bethesda, Maryland, USA). Each sample was repeated three times.

### Western blot

Cells were lysed with RIPA lysate (Beyotime, Shanghai, China), and the concentration of protein was determined by the BCA assay kit (Solarbio, Beijing, China). Briefly, the equal amount of protein was separated with 10% SDS-PAGE (Epizyme, Shanghai, China), and transferred to PVDF membranes (Millipore, MA, USA). The PVDF membranes were blocked with 5% skim milk and incubated at 4 °C overnight with primary antibody, including anti-Fibronectin (Cell Signaling Technology, MA, USA), anti-N-cadherin (CST), anti-E-cadherin (CST), anti-MMP2 (CST), anti-MMP9 (CST), anti-Cytokeratin (Proteintech, Wuhan, China), anti-Vimentin (CST), anti-Ran (Proteintech), anti-Akt (CST), anti-p-Akt (CST), GSK3β (CST), anti-p-GSK3β (CST), anti-β-catenin (CST). β-actin (CST) was used as internal controls. Then the PVDF membranes were incubated with a secondary antibody for 1 h. The blots were scanned by Amersham Imager 600 (GE, Boston, MA, USA). Each sample was repeated three times and the three independent western blot bands were quantitatively analyzed by Image J software (NIH, Bethesda, Maryland, USA).

### RNA-pulldown assay

The pcDNA3.1 plasmid containing WFDC21P or antisense sequence was synthesized by Genomeditech (Shanghai, China). The RNA sequences were linearized with the FastDigest XhoI Kit (ThermoFisher Scientific, Waltham, MA, USA) and T7 MEGAscript Kit (Invitrogen), and transcribed and purified with Purification Kit (TianGen Biotech, Beijing, China). The pulldown assay was performed by using the Desthiobiotinylation Kit and the RNA-Protein Pull Down Kit (ThermoFisher Scientific) based on the manufacturer’s instructions. The proteins were identified with mass spectrometry by the Beijing Institute of Animal Husbandry and Veterinary Medicine.

### RNA immunoprecipitation (RIP)

RIP assay was performed with the RNA Immunoprecipitation Kit (Geneseed, Guangzhou, China) according to the manufacturer’s instructions. Magnetic beads combined with anti-IgG or anti-Ran antibody were incubated with total RNA lysate of HGC-27 cells. The complex was washed from the magnetic beads and the isolated RNA was extracted. The expression of WFDC21P was detected by qRT-PCR.

### Immunohistochemistry (IHC) staining

The IHC staining was conducted with the IHC Kit (Zsgb Bio, Beijing, China). Briefly, sections were incubated with primary and secondary antibodies in sequence and then stained with 3, 3-diaminobenzidine tetrahydrochloride (DAB) and hematoxylin reagent.

We scored the expression of Ran in 20 cases of GC tissues and matched normal tissues by IHC staining. The results of IHC staining were scored by evaluating the extent and intensity of staining in 5 fields of view using a microscope (Olympus, Tokyo, Japan) at ×400 magnification. The staining intensity was divided into four grades: no staining, score 0; pale yellow, score 1; pale brown, score 2; and dark brown, score 3. The positive expression area was also classified into five categories: <5%, score 0; 6–25%, score 1; 26–50%, score 2; 51–75%, score 3; and 76–100%, score 4. The multiplication of intensity and area scores was used as the final Ran expression score. All slides were scored by two independent pathologists from Shandong Provincial Hospital who blind to the clinical data of patients. When there were discrepancies between the two pathologists, the mean score was used.

### Luciferase reporter assay

FOXP3 was selected as a possible transcription factor of WFDC21P based on the Promo and Jaspar database. FOXP3 overexpression plasmid, control plasmid, WFDC21P WT, WFDC21P MT1, and WFDC21P MT2 plasmids were synthesized by Genomeditech (Shanghai, China). HEK-293 cell was transfected with the constructed reporter plasmid. The luminescent signal was detected by Infinite M1000 (Tecan, Männedorf, Switzerland). The detected relative luciferase activity was normalized to the Renilla luciferase activity.

### Tumorigenesis and metastasis in vivo

The 4-weeks-old BALB/c Nude mice were obtained from Charles River (Beijing China) and raised in the SPF (specific-pathogen-free) environment of the Animal Center of Shandong Provincial Hospital. All mice were randomly assigned to each group (*n* = 5 mice per group). In the tumorigenesis assay, luciferase-labeled HGC-27 cells (5 × 10^6^, 150 μl) that stably express sh-WFDC21p and their controls were injected into the right flanks of nude mice. The volume of the tumor was measured every 3 days. After 4 weeks, the mice were killed, and the tumors were dissected and weighed. In the metastasis assay, luciferase-labeled HGC-27 cells (2 × 10^6^, 150 μl) that stably express sh-WFDC21P and their controls were injected into nude mice via tail vein. The bioluminescence of the mice was measured every 3 days by Living-animal Imaging System (LB983 NC100, Berthold, Germany). After 4 weeks, the mice were killed and the lungs were dissected.

All animal experiments were approved by the Committee for Ethics of Animal Experiments of Shandong Provincial Hospital.

### Statistical analysis

All data statistics were carried out via SPSS 26.0 (SPSS, Chicago, IL, USA). Student’s *t*-test or the Mann–Whitney *U* test was used to compare two groups according to the normality evaluation. Paired-samples *t*-test was used to compare the expression of WFDC21P in 57 cancer tissues and paired normal tissues. The Pearson’s correlation analysis was used to analyze the correlations between FOXP3 expression and WFDC21P expression. *χ*^2^ test was used to analyze the correlations between lncRNA WFDC21P expression and clinicopathological variables. Data were shown as mean ± SD. A *p*-value < 0.05 was defined with statistical significance (*p < 0.05, **p < 0.01 and ***p < 0.001).

## Supplementary information

Relative expressions of the selected 10 lncRNAs are shown

Supplementary Figure Legend

The 44 lncRNAs which screened out from the microarray analysis

The top 10 WFDC21P RNA-binding proteins in perceived credibility identified by mass spectrometr

The top ten binding sites with the highest score between FOXP3 and the promoter region of WFDC21P based on Jaspar database

The primer sequences of the genes in this experiment

Oligonucleotide sequences used in the cell transfection

## Data Availability

The data sets used and/or analyzed during the current study are available from the corresponding author on reasonable request.

## References

[1] Sung H, Ferlay J, Siegel R, Laversanne M, Soerjomataram I, Jemal A (2021). Global cancer statistics 2020: GLOBOCAN estimates of incidence and mortality worldwide for 36 cancers in 185 countries. CA Cancer J Clin..

[2] Gao K, Wu J (2019). National trend of gastric cancer mortality in China (2003-2015): a population-based study. Cancer Commun.

[3] Li C, Ye Y, Liang G, Zhang W, Zhang Z, Liu X (2016). Cancer Incidence and Mortality Survey in Wuwei, Gansu Province, Northwestern China from 2003 to 2012: a retrospective population-based study. Chin Med J..

[4] Digklia A, Wagner A (2016). Advanced gastric cancer: current treatment landscape and future perspectives. World J Gastroenterol.

[5] Li T, Gao X, Han L, Yu J, Li H (2018). Identification of hub genes with prognostic values in gastric cancer by bioinformatics analysis. World J Surg Oncol..

[6] Yu C, Chen J, Ma J, Zang L, Dong F, Sun J (2020). Identification of key genes and signaling pathways associated with the progression of gastric cancer. Pathol Oncol Res.

[7] Li Q, He W, Wan G (2020). Methyladenosine modification in RNAs: classification and roles in gastrointestinal cancers. Front Oncol.

[8] Ma L, Bajic V, Zhang Z (2013). On the classification of long non-coding RNAs. RNA Biol.

[9] Sun W, Yang Y, Xu C, Xie Y, Guo J (2016). Roles of long noncoding RNAs in gastric cancer and their clinical applications. J Cancer Res Clin Oncol..

[10] Zhao J, Du P, Cui P, Qin Y, Hu C, Wu J (2018). LncRNA PVT1 promotes angiogenesis via activating the STAT3/VEGFA axis in gastric cancer. Oncogene.

[11] Wei G, Wang X (2017). lncRNA MEG3 inhibit proliferation and metastasis of gastric cancer via p53 signaling pathway. Eur Rev Med Pharmacol Sci..

[12] Sun T, He J, Liang Q, Ren L, Yan T, Yu T (2016). LncRNA GClnc1 promotes gastric carcinogenesis and may act as a modular scaffold of WDR5 and KAT2A complexes to specify the histone modification pattern. Cancer Discov.

[13] Yang Z, Zhi Q, Wang D, Zhang L, Preston B, Brandon C (2016). Long noncoding RNA C21orF96 promotes the migration, invasion and lymph node metastasis in gastric cancer. Anticancer Agents Med Chem.

[14] Guo X, Yang Z, Zhi Q, Wang D, Guo L, Li G (2016). Long noncoding RNA OR3A4 promotes metastasis and tumorigenicity in gastric cancer. Oncotarget.

[15] Kim J, Piao H, Kim B, Yao F, Han Z, Wang Y (2018). Long noncoding RNA MALAT1 suppresses breast cancer metastasis. Nat Genet.

[16] Wang S, Liang K, Hu Q, Li P, Song J, Yang Y (2017). JAK2-binding long noncoding RNA promotes breast cancer brain metastasis. J Clin Invest.

[17] Jin X, Xu X, Jiang Y, Liu Y, Sun W, Guo Y (2019). The endogenous retrovirus-derived long noncoding RNA TROJAN promotes triple-negative breast cancer progression via ZMYND8 degradation. Sci Adv..

[18] Matchett K, McFarlane S, Hamilton S, Eltuhamy Y, Davidson M, Murray J (2014). Ran GTPase in nuclear envelope formation and cancer metastasis. Adv Exp Med Biol..

[19] Boudhraa Z, Carmona E, Provencher D, Mes-Masson A (2020). Ran GTPase: a key player in tumor progression and metastasis. Front Cell Dev Biol.

[20] Ning J, Liu W, Zhang J, Lang Y, Xu S (2013). Ran GTPase induces and enhances invasion in non-small cell lung cancer cells through activation of PI3K-AKT pathway. Oncol Res.

[21] Yuen H, Chan K, Grills C, Murray J, Platt-Higgins A, Eldin O (2012). Ran is a potential therapeutic target for cancer cells with molecular changes associated with activation of the PI3K/Akt/mTORC1 and Ras/MEK/ERK pathways. Clin Cancer Res..

[22] Wang X, Li D, Sun L, Shen G, Liu H, Guo H (2020). Regulation of the small GTPase Ran by miR-802 modulates proliferation and metastasis in colorectal cancer cells. Br J Cancer.

[23] Sheng C, Qiu J, Wang Y, He Z, Wang H, Wang Q (2018). Knockdown of Ran GTPase expression inhibits the proliferation and migration of breast cancer cells. Mol Med Rep.

[24] Zhong Y, Cao L, Ma H, Wang Q, Wei P, Yang J (2020). Lin28A regulates stem-like properties of ovarian cancer cells by enriching RAN and HSBP1 mRNA and up-regulating its protein expression. Int J Biol Sci..

[25] Deng L, Shang Y, Guo S, Liu C, Zhou L, Sun Y (2014). Ran GTPase protein promotes metastasis and invasion in pancreatic cancer by deregulating the expression of AR and CXCR4. Cancer Biol Ther..

[26] Azuma K, Sasada T, Takedatsu H, Shomura H, Koga M, Maeda Y (2004). Ran, a small GTPase gene, encodes cytotoxic T lymphocyte (CTL) epitopes capable of inducing HLA-A33-restricted and tumor-reactive CTLs in cancer patients. Clin Cancer Res.

[27] Zhang L, Li Q, Xu J, Sun G, Xu Z (2020). Cimetidine promotes STUB1-mediated degradation of tumoral FOXP3 by activating PI3K-Akt pathway in gastric cancer. Ann Transl Med..

[28] Zhang L, Xu J, Zhang X, Zhang Y, Wang L, Huang X (2017). The role of tumoral FOXP3 on cell proliferation, migration, and invasion in gastric cancer. Cell Physiol Biochem..

[29] Guo G, He Z, Shi Z (2016). Correlation between FOXP3 expression and gastric cancer. Oncol Lett.

[30] Liu J, Liu Z, Wu Q, Lu Y, Wong C, Miao L (2020). Long noncoding RNA AGPG regulates PFKFB3-mediated tumor glycolytic reprogramming. Nat Commun..

[31] Wang Y, Lu J, Wu Q, Jin Y, Wang D, Chen Y (2019). LncRNA LINRIS stabilizes IGF2BP2 and promotes the aerobic glycolysis in colorectal cancer. Mol Cancer.

[32] Shuai Y, Ma Z, Liu W, Yu T, Yan C, Jiang H (2020). TEAD4 modulated LncRNA MNX1-AS1 contributes to gastric cancer progression partly through suppressing BTG2 and activating BCL2. Mol Cancer.

