# Supplementary figures and images for: A new candidate oncogenic lncRNA derived from pseudogene WFDC21P promotes tumor progression in gastric cancer
Source: Cell Death Dis. 2021 Oct 2;12(10):903. doi: 10.1038/s41419-021-04200-x (PMC8487428; doi:10.1038/s41419-021-04200-x)

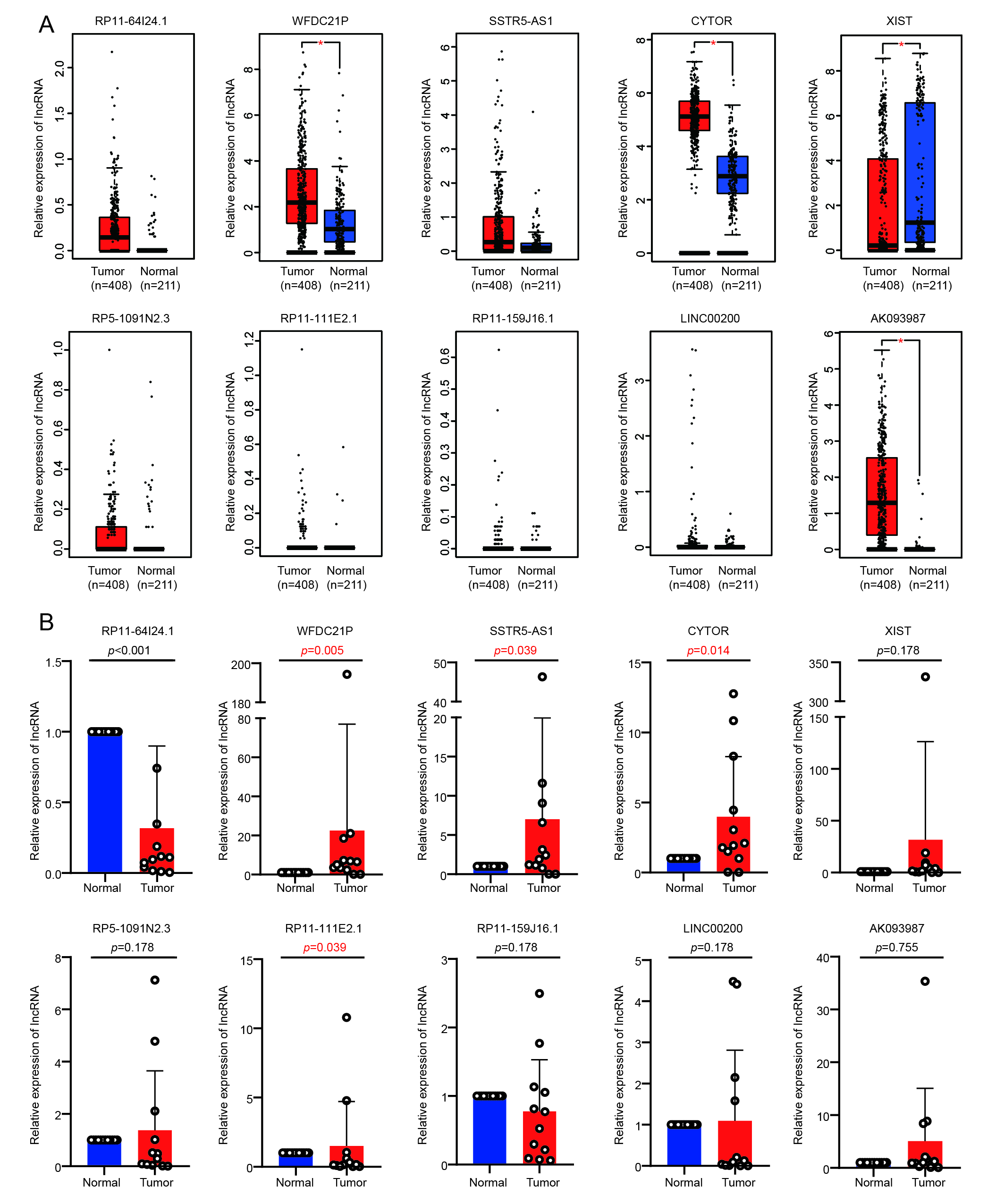

Supplement: Supplementary file 1 — Relative expressions of the selected 10 lncRNAs are shown [file 41419_2021_4200_MOESM1_ESM.tif]
