# Supplementary material for: A new candidate oncogenic lncRNA derived from pseudogene WFDC21P promotes tumor progression in gastric cancer
Source: Cell Death Dis. 2021 Oct 2;12(10):903. doi: 10.1038/s41419-021-04200-x (PMC8487428; doi:10.1038/s41419-021-04200-x)
Supplement: Supplementary file 2 — Supplementary Figure Legend [file 41419_2021_4200_MOESM2_ESM.docx]

**Supplementary Fig. 1 Relative expressions of the selected 10 lncRNAs are shown. A.** Relative expressions of the selected 10 lncRNAs in GC tissues and normal tissues from TCGA. **B**. Relative expressions of the selected 10 lncRNAs in 12 GC tissues and paired normal tissues from Shandong Provincial Hospital were detected by qRT-PCR (Mann-Whitney *U* test).
