# Supplementary material for: A new candidate oncogenic lncRNA derived from pseudogene WFDC21P promotes tumor progression in gastric cancer
Source: Cell Death Dis. 2021 Oct 2;12(10):903. doi: 10.1038/s41419-021-04200-x (PMC8487428; doi:10.1038/s41419-021-04200-x)
Supplement: Supplementary file 3 — The 44 lncRNAs which screened out from the microarray analysis [file 41419_2021_4200_MOESM3_ESM.docx]

Supplementary Table 1:

The 44 lncRNAs which screened out from the microarray analysis

| Gene Name | T1 | T2 | T3 | N | Fold change | Length |
| --- | --- | --- | --- | --- | --- | --- |
| RP11-64I24.1 | 1179.3018 | 1308.7886 | 687.23047 | 187.13702 | 6.3926306 | 270 |
| WFDC21P | 990.63416 | 1537.5171 | 490.8844 | 164.389 | 6.5019283 | 621 |
| SSTR5-AS1 | 1214.6047 | 1445.2618 | 29.71988 | 59.113884 | 6.7763014 | 2948 |
| CYTOR | 984.7203 | 1441.7675 | 936.6458 | 186.26361 | 6.896078 | 518 |
| XIST | 7.6560993 | 9.740136 | 3151.2322 | 6.775211 | 6.907764 | 446 |
| RP5-1091N2.3 | 799.08453 | 1144.2395 | 45.592587 | 50.80917 | 7.5051765 | 375 |
| RP11-111E2.1 | 1672.0753 | 1961.8496 | 85.211105 | 95.05469 | 7.803054 | 384 |
| RP11-159J16.1 | 919.67737 | 1532.1348 | 848.72797 | 153.2226 | 8.097827 | 1150 |
| RPSAP1 | 6366.1006 | 9526.783 | 7468.846 | 1043.8236 | 8.280375 | 880 |
| AC016737.1 | 5165.8403 | 6777.1113 | 5728.8306 | 787.84656 | 8.460522 | 873 |
| AC002069.5 | 2848.0872 | 3648.286 | 7899.721 | 590.69446 | 8.491534 | 1489 |
| RP11-346A3.2 | 3667.8264 | 5304.037 | 4999.8604 | 610.69885 | 8.654079 | 862 |
| RP11-402K9.2 | 5118.1826 | 6621.3438 | 6443.877 | 716.80383 | 9.552072 | 892 |
| RP11-240M16.2 | 4232.196 | 4968.0776 | 3658.2476 | 498.91833 | 9.779629 | 878 |
| RP4-781L3.1 | 12459.154 | 18861.758 | 6113.6123 | 1286.7367 | 9.866653 | 402 |
| AC097639.8 | 6160.122 | 9226.27 | 7747.6665 | 821.35596 | 10.449097 | 881 |
| HLA-DRB6 | 18406.902 | 24389.986 | 14191.954 | 1944.3917 | 10.6923895 | 1235 |
| RP5-1172A22.1 | 173.28937 | 251.73573 | 33.89003 | 8.2074 | 10.881379 | 637 |
| APOC1P1 | 1307.9089 | 1812.8241 | 320.57565 | 95.41349 | 11.021978 | 939 |
| AC090042.1 | 1375.4725 | 1388.338 | 1370.7128 | 138.98178 | 11.429553 | 405 |
| RP11-325M4.1 | 229.75784 | 238.40373 | 46.542778 | 10.161129 | 11.65983 | 1284 |
| RP11-336N8.4 | 4694.614 | 6734.209 | 3532.508 | 466.5706 | 11.732215 | 238 |
| RP11-162K6.1 | 1222.4801 | 1732.013 | 1887.5216 | 152.87488 | 12.086472 | 883 |
| RP11-488L18.4 | 625.7514 | 957.2658 | 44.626915 | 25.799547 | 12.653702 | 1465 |
| RP5-1100H13.3 | 265.6426 | 248.83792 | 150.8154 | 18.424288 | 12.750518 | 564 |
| RP11-288G3.2 | 1415.7062 | 1913.7725 | 64.384636 | 46.60281 | 13.211287 | 1501 |
| RP11-85F14.1 | 203.15306 | 144.90324 | 144.358 | 10.4011 | 14.14843 | 1002 |
| RP11-600F24.1 | 435.78717 | 215.05905 | 459.86392 | 27.67161 | 14.534102 | 884 |
| LOC100133669 | 154.1967 | 204.33774 | 82.13484 | 6.7209673 | 15.088175 | 709 |
| HLA-DRB9 | 13432.018 | 13955.776 | 8436.606 | 882.90753 | 15.12837 | 343 |
| PTTG3P | 6766.3457 | 10079.891 | 5026.4805 | 470.37775 | 16.816404 | 609 |
| RP11-27G9.1 | 1462.4713 | 1397.9755 | 52.554195 | 27.635763 | 18.551308 | 630 |
| RP11-111F10.2 | 1547.8657 | 1701.1292 | 34.01159 | 23.996698 | 19.744358 | 873 |
| RP11-411K7.4 | 36612.156 | 13.08641 | 54.63974 | 18.141472 | 19.88931 | 544 |
| BC084558 | 713.6277 | 778.82635 | 6.2748013 | 5.8031325 | 20.340866 | 1010 |
| AC005822.1 | 814.1078 | 995.9276 | 6.6662283 | 6.552504 | 21.564194 | 1034 |
| RP11-45J1.1 | 3525.246 | 5339.1904 | 2661.5938 | 190.62645 | 22.2159 | 400 |
| RP11-738E22.1 | 4327.703 | 6473.1387 | 4625.9756 | 230.07571 | 25.28395 | 450 |
| AC018634.9 | 187.95886 | 262.55298 | 394.3555 | 6.6851087 | 30.006332 | 401 |
| RP11-156J23.1 | 766.5876 | 1195.5615 | 2537.8918 | 47.144035 | 31.497276 | 453 |
| RP11-713C19.1 | 1104.5912 | 1608.6143 | 1204.6879 | 41.75161 | 34.507523 | 357 |
| RP11-190J1.3 | 107.91651 | 1883.8507 | 151.33556 | 5.7761626 | 37.2264 | 291 |
| NCRNA00200 | 174.30739 | 246.68301 | 1048.814 | 7.4459577 | 37.474087 | 2434 |
| AK093987 | 975.45703 | 1324.8467 | 84.765205 | 5.931316 | 54.96785 | 2285 |
