# Supplementary material for: A new candidate oncogenic lncRNA derived from pseudogene WFDC21P promotes tumor progression in gastric cancer
Source: Cell Death Dis. 2021 Oct 2;12(10):903. doi: 10.1038/s41419-021-04200-x (PMC8487428; doi:10.1038/s41419-021-04200-x)
Supplement: Supplementary file 4 — The top 10 WFDC21P RNA-binding proteins in perceived credibility identified by mass spectrometr [file 41419_2021_4200_MOESM4_ESM.docx]

Supplementary Table 2:

The top 10 WFDC21P RNA-binding proteins in perceived credibility identified by mass spectrometry

| Accession | Gene Name | Coverage (%) | PSMs | Unique Peptides | Molecular Weight (kDa) | calc. pI | Score Sequest HT: Sequest HT |
| --- | --- | --- | --- | --- | --- | --- | --- |
| B5MDF5 | GTPase Ran | 5 | 2 | 1 | 26.2 | 7.01 | 5.42 |
| F6RFD5 | DSTN | 10 | 2 | 1 | 15.4 | 8.59 | 5.39 |
| P04264 | KRT1 | 6 | 4 | 3 | 66 | 8.12 | 4.8 |
| P23528 | CFL1 | 8 | 2 | 1 | 18.5 | 8.09 | 3.54 |
| B8ZZQ6 | PTMA | 14 | 2 | 1 | 11.8 | 3.81 | 3.24 |
| H0Y9Y4 | RPS3A | 6 | 2 | 1 | 23.5 | 9.86 | 2.5 |
| E9PKE3 | HSPA8 | 4 | 1 | 1 | 68.8 | 5.52 | 2.33 |
| P13645 | KRT10 | 2 | 1 | 1 | 58.8 | 5.21 | 2.3 |
| E9PD92 | G6PD | 4 | 1 | 1 | 29.5 | 7.17 | 2.14 |
| Q01469 | FABP5 | 7 | 1 | 1 | 15.2 | 7.01 | 2.13 |
