# Supplementary material for: A new candidate oncogenic lncRNA derived from pseudogene WFDC21P promotes tumor progression in gastric cancer
Source: Cell Death Dis. 2021 Oct 2;12(10):903. doi: 10.1038/s41419-021-04200-x (PMC8487428; doi:10.1038/s41419-021-04200-x)
Supplement: Supplementary file 5 — The top ten binding sites with the highest score between FOXP3 and the promoter region of WFDC21P based on Jaspar database [file 41419_2021_4200_MOESM5_ESM.docx]

Supplementary Table 3:

The top ten binding sites with the highest score between FOXP3 and the promoter region of WFDC21P based on Jaspar database

| **Number** | **Name** | **Sequence ID** | **Start** | **End** | **Strand** | **Predicted sequence** | **Score** |
| --- | --- | --- | --- | --- | --- | --- | --- |
| 1 | FOXP3 | WFDC21P | 710 | 716 | - | GCAAACA | 9.35863 |
| 2 | FOXP3 | WFDC21P | 1179 | 1185 | - | GTAACCA | 7.79684 |
| 3 | FOXP3 | WFDC21P | 1087 | 1093 | + | AAAAACA | 7.28008 |
| 4 | FOXP3 | WFDC21P | 1338 | 1344 | + | GCAAAGA | 6.77879 |
| 5 | FOXP3 | WFDC21P | 1696 | 1702 | + | GTTAATA | 6.35664 |
| 6 | FOXP3 | WFDC21P | 1681 | 1687 | + | ATATACA | 6.27724 |
| 7 | FOXP3 | WFDC21P | 1105 | 1111 | - | TTAAACA | 6.14419 |
| 8 | FOXP3 | WFDC21P | 598 | 604 | + | ATAAGTA | 6.1014 |
| 9 | FOXP3 | WFDC21P | 1651 | 1657 | - | ATAAGTA | 6.1014 |
| 10 | FOXP3 | WFDC21P | 939 | 945 | - | ATAAGGA | 5.84475 |
