# Supplementary material for: A new candidate oncogenic lncRNA derived from pseudogene WFDC21P promotes tumor progression in gastric cancer
Source: Cell Death Dis. 2021 Oct 2;12(10):903. doi: 10.1038/s41419-021-04200-x (PMC8487428; doi:10.1038/s41419-021-04200-x)
Supplement: Supplementary file 6 — The primer sequences of the genes in this experiment [file 41419_2021_4200_MOESM6_ESM.docx]

Supplementary Table 4:

The primer sequences of the genes in this experiment

| Gene | Primer sequence |
| --- | --- |
| WFDC21P | F:GTGTTGCAGGGTAAGGACAGG |
|  | R:TCCACAGGCTTGCTGTTTTCA |
| GTPase Ran | F:CATCCCCATTGTGTTGTGTGG |
|  | R:GGGTCTCCAATGAGCTTCCT |
| FOXP3 | F:AAGTTCCACAACATGCGACC |
|  | R:CATTGAGTGTCCGCTGCTTC |
| β-actin | F:CATGTACGTTGCTATCCAGGC |
|  | R:CTCCTTAATGTCACGCACGAT |
