# Supplementary material for: A new candidate oncogenic lncRNA derived from pseudogene WFDC21P promotes tumor progression in gastric cancer
Source: Cell Death Dis. 2021 Oct 2;12(10):903. doi: 10.1038/s41419-021-04200-x (PMC8487428; doi:10.1038/s41419-021-04200-x)
Supplement: Supplementary file 7 — Oligonucleotide sequences used in the cell transfection [file 41419_2021_4200_MOESM7_ESM.docx]

Supplementary Table 5:

Oligonucleotide sequences used in the cell transfection

| Groups | Oligonucleotide sequences |
| --- | --- |
| sh-WFDC21P | GGAUCAUCUUUCCCUAAGA |
| si-Ran | CCAUUGUCUUCCACCGAAA |
| si-FOXP3 | GCACAUUCCCAGAGUUCCU |
| si/sh-NC | UUCUCCGAACGUGUCACGU |
